# Posterior pharyngeal wall augmentation in post-adenoidectomy velopharyngeal insufficiency

**DOI:** 10.1007/s00405-022-07406-7

**Published:** 2022-05-09

**Authors:** Ayman Amer, Anas Magdy Saqr, Ahmed Mohamed Zayed, Mohamed El-Kotb, Ahmed Elsobki

**Affiliations:** 1 Phoniatric Unit, ORL Department, Faculty of Medicine, Mansoura University, Mansoura, Egypt; 2 ORL Department, Mansoura Insurance Hospital, Mansoura, Egypt; 3 ORL Department, Faculty of Medicine, Mansoura University, Mansoura, Egypt

**Keywords:** VPI, Augmentation, Post-adenoidectomy, Cartilage, Speech

## Abstract

**Purpose:**

To assess the efficacy of posterior pharyngeal wall augmentation using septal or conchal cartilages with other bulks—according to the persistent gap and the individual anatomy of each patient—in improving velopharyngeal function in patients who acquired persistent velopharyngeal insufficiency (VPI) post-adenoidectomy.

**Methods:**

Observational descriptive prospective case series of 24 patients (their ages ranged between 3 and 26 years) who developed persistent VPI post-adenoidectomy (more than 3 months) although they had normal speech resonance before adenoidectomy.

**Results:**

The present study demonstrated that statistically significant improvement in auditory perceptual assessment (APA) was found regarding all obligatory speech disorders and unintelligibility of speech. Significant improvement was observed in the degree of velar mobility, size of the persistent gap, and the gap distance between velum and posterior pharyngeal wall at rest and during phonation in post-operative evaluation versus pre-operative. A significant change was observed in the closure pattern of the velopharyngeal port (VPP) as all patients turned to coronal closure.

**Conclusions:**

Posterior pharyngeal wall augmentation could be used in VPI post-adenoidectomy up to 7 mm and lead to better speech outcomes. Also, it revealed that using conchal and/or septal cartilage as a graft regardless of the patient’s age is a safe procedure.

## Introduction

Velopharyngeal insufficiency (VPI) means failure of the soft palate, lateral and posterior pharyngeal walls to make a seal between the mouth cavity and the nasal cavity throughout speech [1]. Adenoidectomy is a well-known cause of VPI. It is a condition characterized by hypernasality, nasal air emission, consonant imprecision, and in some cases, nasal regurgitation of fluids. It is hard to establish its exact incidence, but it has been estimated between one in 1500 and one in 10,000 adenoidectomies. It is often due to the unmasking of a pre-existing palatal problem by removal of the tissue, a poorly functioning palate was achieving nasopharyngeal closure against it, such as the occult, submucous cleft, velopharyngeal (VP) disproportion, and poor palatal mobility. Post-adenotonsillectomy scarring of the anterior pillar or irregular posterior pharyngeal wall is another cause [2].

Surgery is the definitive treatment of VPI aiming to separate the nasopharynx from the oropharynx during speech and swallowing to diminish nasal airflow during the speech while maintaining upper airway patency [3]. Augmentation pharyngoplasty, in which the posterior nasopharynx is augmented by tissue filler or grafts, is an approach that holds great promise for correcting VPI [4].

The target is to enhance the connection among the velum and the posterior pharyngeal wall during velopharyngeal closure. The idea comprises front displacing of the posterior pharyngeal wall through which it delivers a more accessible connecting spot for the soft palate, permitting a proper velopharyngeal closure and avoiding leakage of nasal air during oral phonemes. Various techniques have been described, with the classical approach being a superiorly based rolled pharyngeal flap. More recently, augmentation by direct implantation through an incision or injection into the posterior pharyngeal wall has been described. Autologous and non-autologous materials are attainable and described, such as cartilage, fat, fascia, silicone, non-cellular dermis, polytetrafluoroethylene, and calcium hydroxyapatite [5].

This work aimed to assess the efficacy of posterior pharyngeal wall augmentation using septal or conchal cartilages with other bulks—according to the persistent gap and each patient’s anatomy in improving velopharyngeal function in patients who acquired persistent VPI post-adenoidectomy.

## Subjects and methods

### Subjects

This observational descriptive prospective case-series study was conducted on 24 surgically fit patients who developed velopharyngeal insufficiency due to persistent post-adenoidectomy gap during speech. The patients were selected from otorhinolaryngology and phoniatric outpatient clinics during the period from October 2019 to September 2020. Their ages ranged between 3 and 26 years.

### Inclusion criteria

Patients who developed persistent VPI post-adenoidectomy (more than 3 months) although they had normal speech resonance before adenoidectomy.

### Exclusion criteria

1. Patients who had VPI before adenoidectomy.
2. Patients who developed VPI post-adenoidectomy with spontaneous recovery 3 months after the operation.
3. Patients who had VPI due to a cause rather than adenoidectomy.
4. Patients who developed Velopharyngeal dysfunction (VPD) post-adenoidectomy with stimulability were treated by speech therapy.

### Methods

All patients were subjected to the following protocol of assessment:

1. History taking: Asking for the duration after adenoidectomy and symptoms of VPI post-adenoidectomy as nasality and regurgitation of food from the nose.
2. Vocal tract examination: including examination of the nose, lip, alveolus, palate, velar length, and mobility, looking for scarring of the velum post-tonsillectomy and searching for signs of submucous cleft palate (bifid uvula, zona blucida, and grooving at the posterior nasal spine).
3. Clinical diagnostic aids: All the patients were assessed pre- and post-surgery by the same phoniatrician using the following diagnostic aids:

#### Documentation of auditory perceptual assessment (APA)

It was done using high-fidelity speech and voice recording in a soundproof room. Then ask the patient to sit down comfortably facing the microphone with a 10 cm distance from it and with comfortable, average loudness; the patient is asked to repeat the following protocol of speech and voice: name of the patient, standard passage, and counting from 1 to 10. Then the recorded material is assessed by the phoniatrician, and the speech disorders were divided into obligatory speech disorders, compensatory speech disorders, and unintelligibility of speech. Obligatory speech disorders include the type and degree of nasality (from 0 to IV = no, slight, mild, moderate, severe), degree of consonant imprecision (from 0 to IV), and nasal emission of air (present or absent). Compensatory speech disorders include glottal articulation (present or absent), pharyngeal articulation (present or absent), and facial grimace (present or absent). The unintelligibility of speech: graded from 0 to IV, either due to obligatory speech disorders or compensatory speech disorders, or both.

#### Documentation of visual assessment of velopharyngeal port (VPP)

Through multiview videofluoroscopy that was performed by 2 phoniatricians in the fluoroscopic unit, radiology department, and 1 ml thick barium was applied through the nasal cavity using a dropper (pipette) to contrast soft tissues against the surrounding skeletal structures. Fluoroscopic views were obtained in the lateral, anteroposterior (frontal), and Towne’s positions (the patient was erect, and the head was deflected downwards with the chin closer to the chest), while the subjects were repeating specific speech samples. In lateral view, the gap distance between the velum and the posterior pharyngeal wall was measured at rest and during phonation of the sustained/a/vowel by the internal ruler of the fluoroscopic unit, velar length concerning the posterior pharyngeal wall, and the degree of velar mobility graded from 0 to IV (0 = no movement and IV = complete closure). In frontal view, the following parameters were graded: degree of movement of the lateral pharyngeal walls from 0, which represents: no movement, to 4, which represents: complete closure, symmetry of the lateral pharyngeal wall movement (0 = asymmetrical and 1 = symmetrical), and the gap distance between the lateral pharyngeal walls were measured at rest and during phonation of the sustained/a/vowel. The following were assessed during Towne’s view: movement of the velum and lateral pharyngeal walls, closure pattern, and presence of persistent velopharyngeal gap during phonation.

### Surgical interference

Augmentation pharyngoplasty operation was done for all the patients. Septal cartilage was only used for seven adult patients. Conchal cartilage only was used for 13 patients. Conchal cartilage was taken from only one auricle except for three patients; the graft was taken from both auricles. A combination of septal and conchal cartilage was needed for four patients. The thickness of different cartilage bulks were determined according to the measured gap between the velum and the posterior pharyngeal wall during phonation, the gap size was determined by 2 phoniatricians during assessment of velopharyngeal port by lateral view videofluoroscopy.

**Fig. 1:**
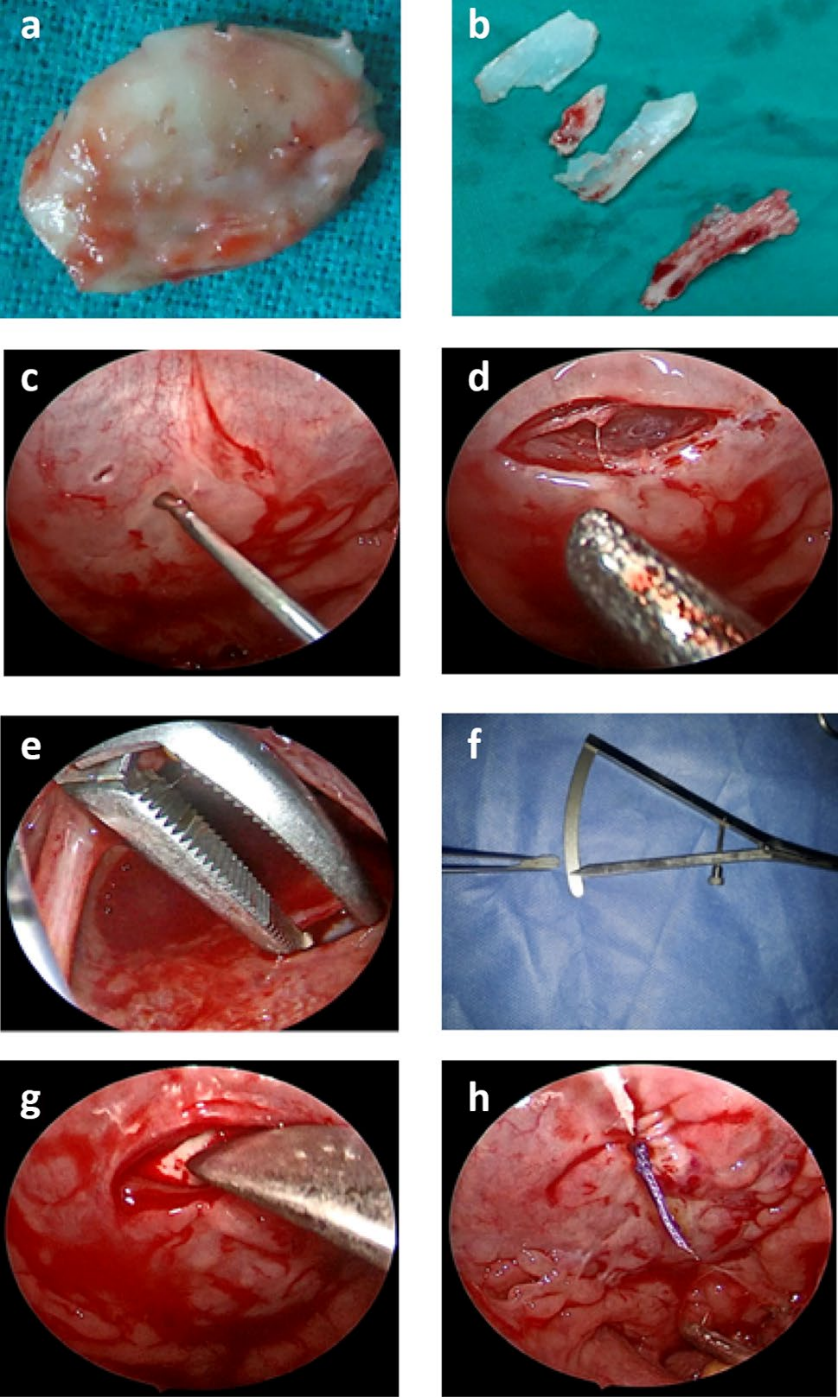
Operation steps **a** Conchal cartilage graft. **b** Septal cartilage graft. **c** Injection. **d** Transverse incision at the posterior wall of the nasopharynx. **e** Dissection and pocket formation. **f** Compass for detection of cartilage thickness. **g** Cartilage at the pocket. **h** Closed wound

The operation steps (Fig. 1):

1. Cartilage graft was taken (septal and/or conchal).
2. The retractor and tongue blade (Boyle Davis) were adjusted to completely expose the palatopharyngeus folds in the operating field. The soft palate was retracted superiorly to expose the posterior pharyngeal wall.
3. 1\100,000 adrenaline was injected at the site of incision, which was detected according to the level of closure of the VPP during phonation determined by videofluoroscopy.
4. Transverse incision was taken at the posterior wall of the nasopharynx superficial to the prevertebral fascia.
5. Dissection and pocket formation superficial to the prevertebral fascia were done.
6. A compass detected the cartilage thickness by putting it in layers.
7. The cartilage was put in the pocket.
8. The wound was closed by stitches.

### Post-operative care

- All patients were discharged home on the day of surgery.
- Post-operative antibiotics were prescribed.
- Patients return for post-operative evaluation of the graft site:
  - Removal of stitches at conchal wound after 1 week.
  - Removal of the septal stent after 2 weeks.
- Patients return for Phoniatrics post-operative evaluation after 1 month.

Speech therapy was needed for some patients to correct compensatory speech disorders that are not corrected by the surgical intervention.

The study was explained to all participants, and informed written consent was obtained from adults and the parents of children before the start of the study.

### Statistical analysis

The collected data was revised, coded, tabulated, and introduced to a PC using Statistical Package for Social Science (IBM Corp. Released in 2011. IBM SPSS Statistics for Windows, Version 20.0. Armonk, NY: IBM Corp.). Descriptive statistics were calculated in the form of Mean ± Standard deviation (SD), median and range (minimum–maximum), and frequency (number-percent). For continuous data, the Shapiro–Wilk test was used to assess the normality of the studied variables with a *p* value < 0.05, indicating non-normally distributed data. Analytical statistics: in the statistical comparison between the different groups, the significance of difference was tested using one of the following tests: student’s *t* test (paired): used to compare the mean of two related groups of normally distributed data. Wilcoxon signed-rank test: used to compare two related groups of numerical (non-normally distributed data). McNemar and Stuart–Maxwell tests were used to compare pre- and post-results of a categorical variable (McNemar test for dichotomous variable and Stuart–Maxwell test for ordinal variables). Spearman’s correlation coefficient test was used correlating different parameters. *p* value < 0.05 was considered statistically significant.

## Results

### Descriptive and comparative statistics

#### Description of demographic data of studied group

The demographic data are shown in Table 1.

**Table 1:** Socio-demographic characteristics and duration after adenoidectomy among studied cases

	Median	Range
Age (in years)	8.00	3.00–26.00
Duration after adenoidectomy (in years)	2.50	0.75–13.00

	No	%
Sex		
Male	12	50.0
Female	12	50.0

Data expressed as median (range) or as frequency (number-percent)

#### Pre- versus post-assessment of APA items

Description and comparison of the variables of APA items in pre- and post-augmentation are summarized in Table 2. Statistically significant improvements were demonstrated regarding all obligatory speech disorders and unintelligibility of speech.

**Table 2:** Pre- versus post-assessment of APA items (obligatory speech disorders/compensatory speech disorders/unintelligibility of speech)

		Pre		Post		McNemar/Stuart–Maxwell test
		No	%	No	%	*p* value
Obligatory speech disorders						
Degree of open nasality						
Grade 0		0	0.0	9	37.5	< 0.001*^,a^
Grade I		0	0.0	9	37.5	
Grade II		12	50.0	6	25.0	
Grade III		12	50.0	0	0.0	
Consonant imprecision						
Grade 0		0	0.0	6	25.0	< 0.001*^,a^
Grade I		6	25.0	6	25.0	
Grade II		6	25.0	12	50.0	
Grade III		12	50.0	0	0.0	
Nasal air emission						
Absent		3	12.5	18	75.0	< 0.001*^,b^
Present		21	87.5	6	25.0	
Compensatory speech disorders						
Facial grimace						
Absent		24	100	24	100	1.00^b^
Present		0	0	0	0	
Glottal articulation						
Absent		18	75	18	75	1.00^b^
Present		6	25	6	25	
Pharyngealization of fricatives						
Absent		21	87.5	21	87.5	1.00 ^b^
Present		3	12.5	3	12.5	
Unintelligibility of speech						
Grade 0		0	0	15	62.5	< 0.001*^,b^
Grade I		3	12.5	6	25	
Grade II		21	87.5	3	12.5	

Data expressed as frequency (number-percent)

*Significance test used

^a^McNemar test

^b^Stuart–Maxwell test

#### Pre- versus post-assessment of velopharyngeal port parameters obtained from videofluoroscopy

Significant improvement was observed in the degree of velar mobility, the pattern of closure, and the absence of persistent gap during phonation in post-operative evaluation versus pre-operative, as shown in Table 3.

**Table 3:** Pre- versus post-assessment of velopharyngeal port parameters obtained from videofluoroscopy

	Pre		Post		McNemar/Stuart–Maxwell test
	No	%	No	%	*p* value
Degree of Velar mobility					
Grade I	0	0.0	0	0.0	< 0.001*^,b^
Grade II	9	37.5	0	0.0	
Grade III	15	62.5	12	50.0	
Grade IV	0	0.0	12	50.0	
Lateral pharyngeal wall mobility					
Grade I	9	37.5	9	37.5	1.00^b^
Grade II	15	62.5	15	62.5	
Grade III	0	0.0	0	0.0	
Grade IV	0	0.0	0	0.0	
Pattern of closure					
Circular	9	37.5	0	0.0	0.002^a^
Coronal	15	62.5	24	100.0	
Persistent gap					
Absent	0	0.0	21	87.5	< 0.001*^,a^
Persistent	24	100.0	3	12.5	

Data expressed as frequency (number-percent) test used

*Statistically significant

^a^McNemar test

^b^Stuart–Maxwell test

#### Pre- versus post-assessment of velopharyngeal gap distance obtained from Videofluoroscopy:

**Fig. 2:**
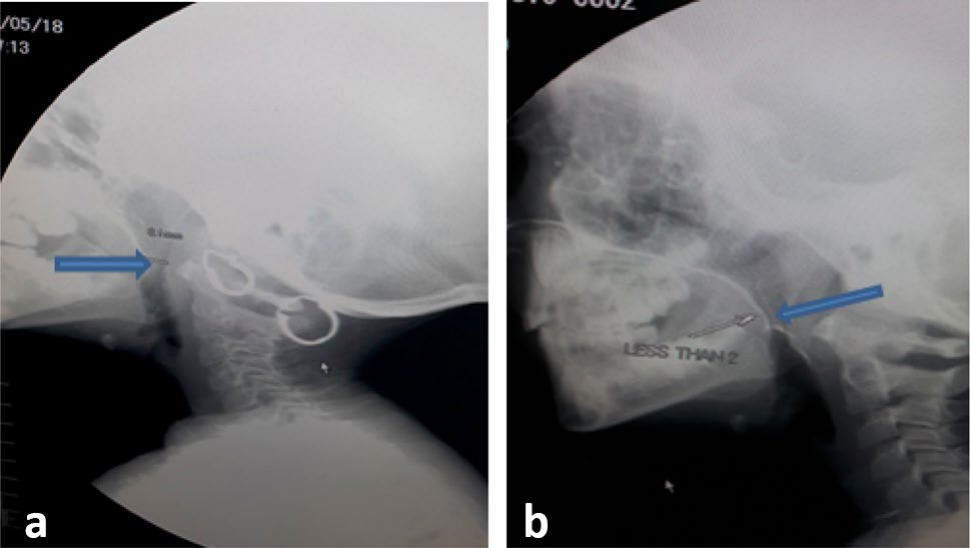
Videofluroscopy during phonation of/a/sound **a** pre-augmentation showing persistent gap. **b** Post-augmentation showing complete closure (the arrows refer to the velopharyngeal closure)

**Fig. 3:**
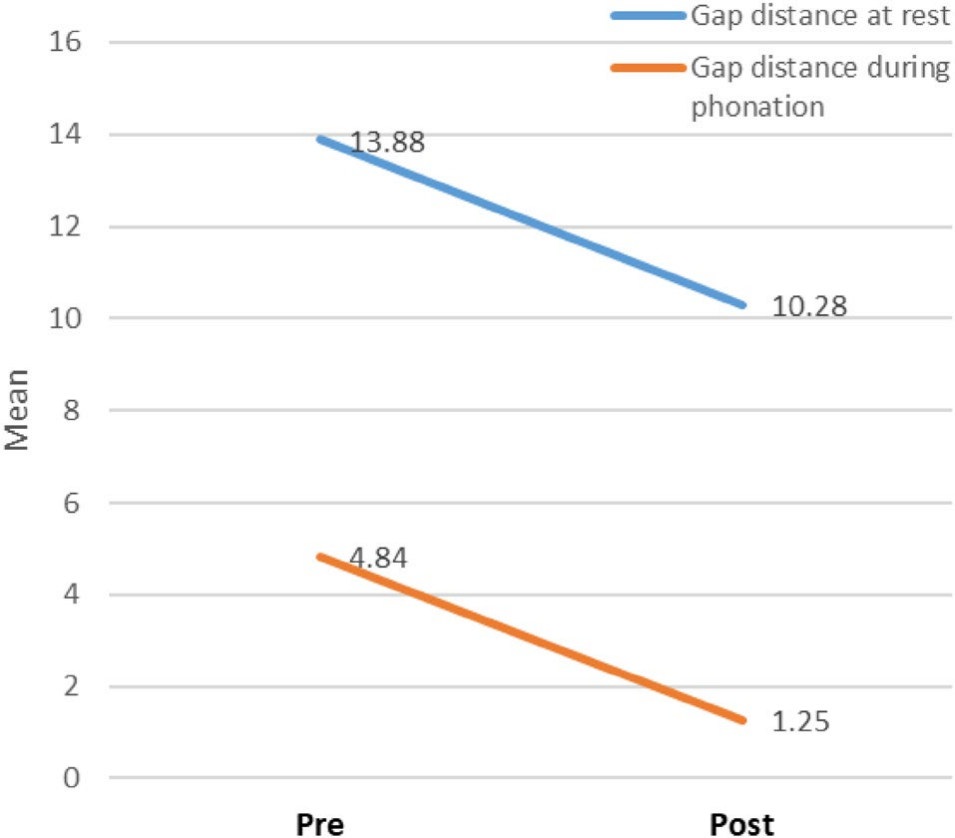
Line graph showing gap distance change pre- and post-operative

Significant improvement was noticed in gap distance between velum and posterior pharyngeal wall at rest and during phonation in post-test assessment than pretest (Table 4, Figs. 2, 3).

**Table 4:** Pre- versus post-test assessment of gap distance (mm) between the velum and post-pharyngeal wall at rest and during phonation

	Pre		Post		*t*/*Z* test
Gap distance at rest (mean ± SD)	13.88 ± 3.23		10.28 ± 2.81		< 0.001*^,a^
Gap distance during phonation (median-range)	4.85	3.00–7.00	1.00	0.00–2.80	< 0.001*^,b^
Mean ± SD	4.84 ± 1.25		1.25 ± 1.12		

Data expressed as mean ± SD or as median (range)

*SD* standard deviation

*Significance < 0.05

^a^Paired *t* test

^b^Wilcoxon signed rank test

### Correlative statistics

1. Age and gap measurements

**Fig. 4:**
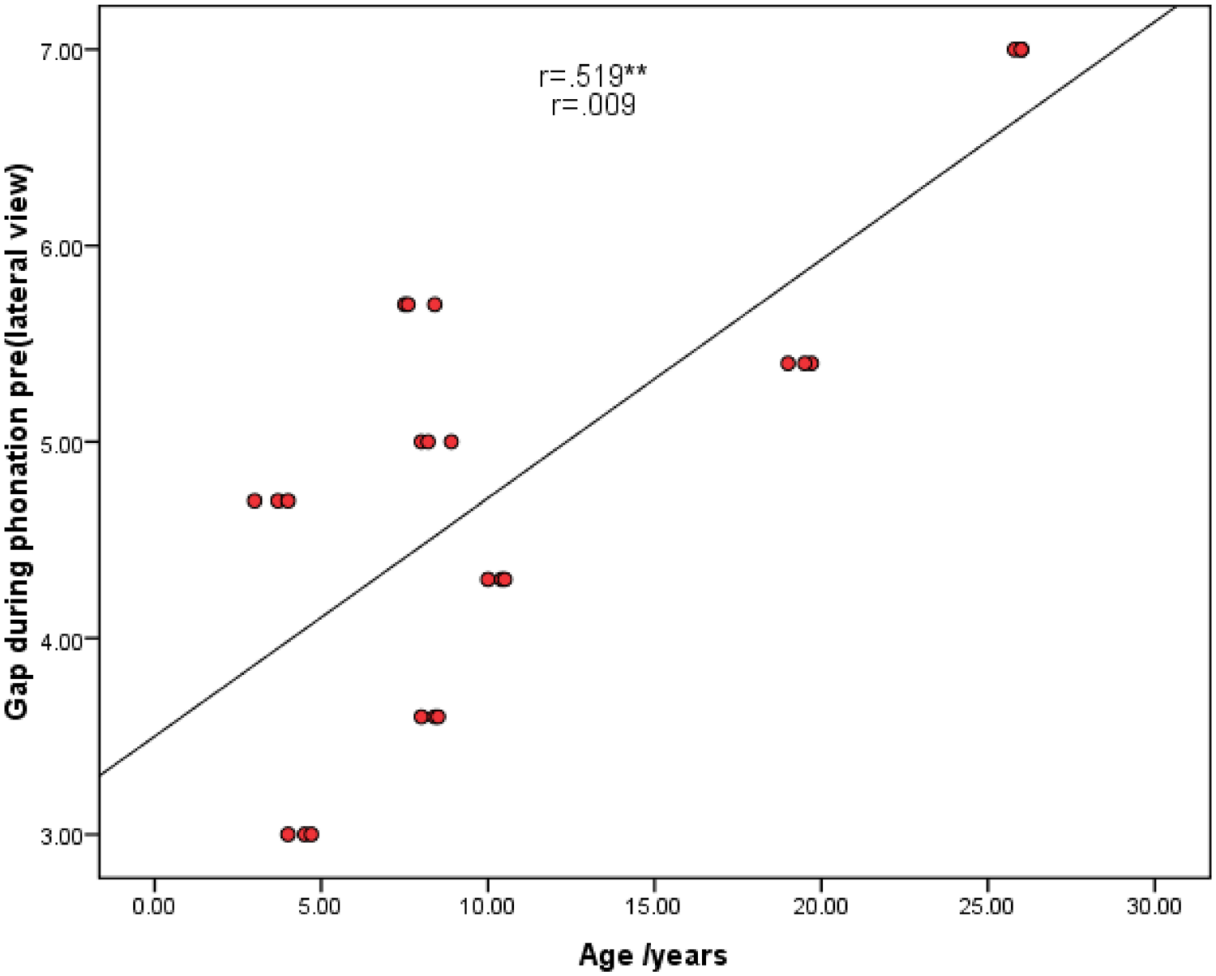
Scatter diagram showing the statistically significant positive correlation between age and gap measurements during phonation pre-operative (lateral view)

**Fig. 5:**
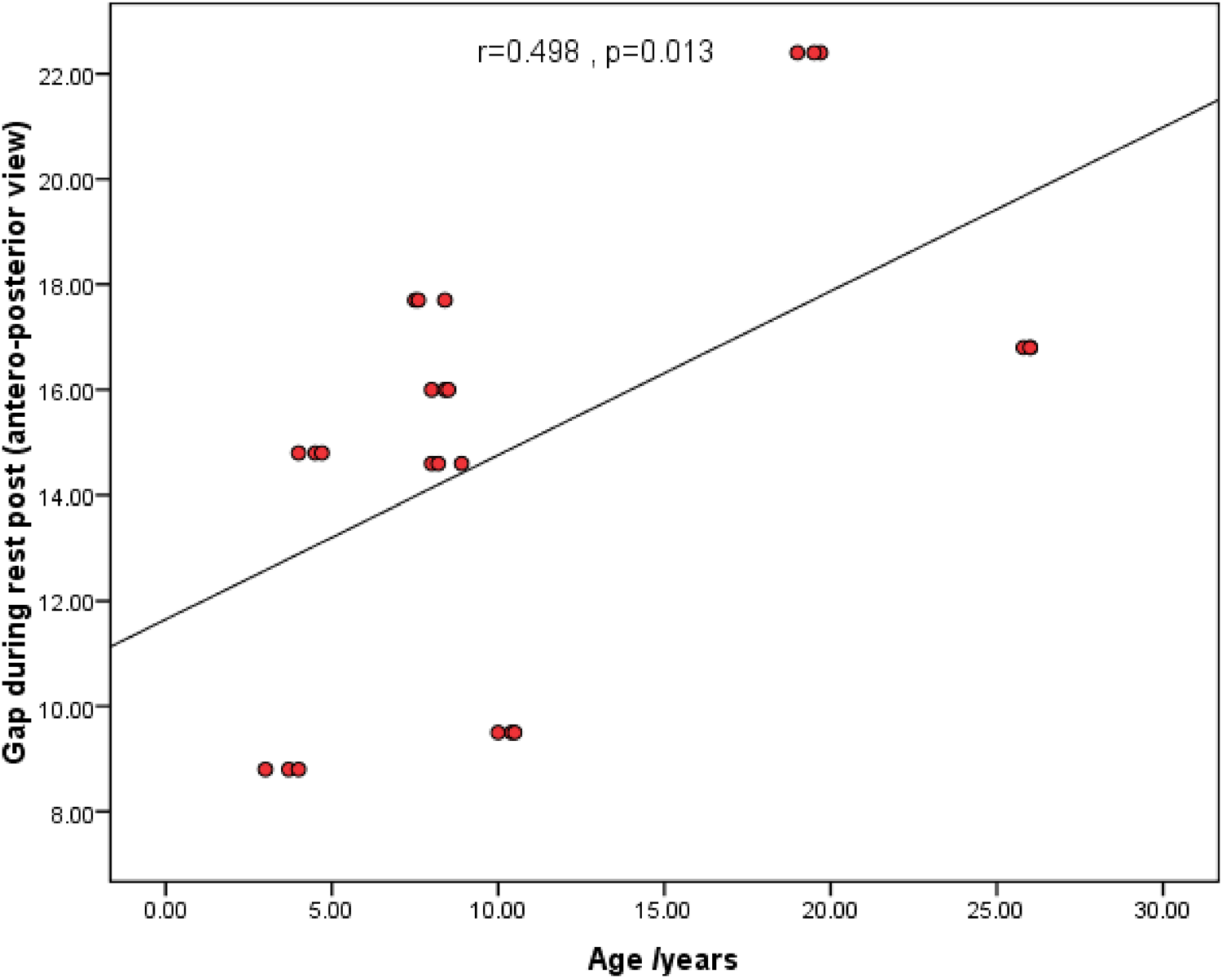
Scatter diagram showing the statistically significant positive correlation between age and gap measurements during rest post-operative (anteroposterior view)

**Fig. 6:**
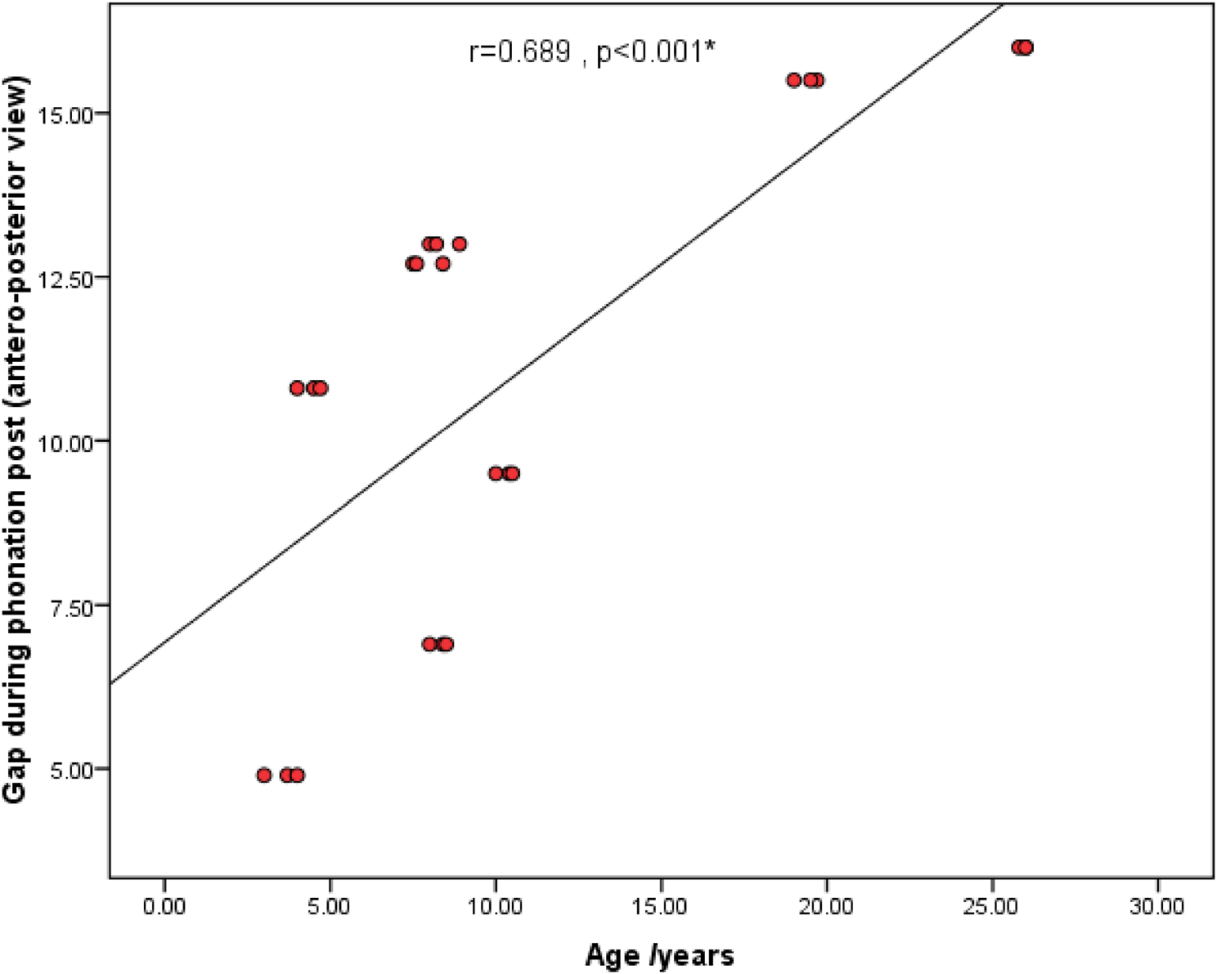
Scatter diagram showing the statistically significant positive correlation between age and gap measurements during phonation post-operative (anteroposterior view)

There was a statistically significant positive correlation between age and gap measurements during phonation pre-operative (lateral view), while post-operatively, a statistically significant positive correlation was between age and gap measurements during rest and during phonation (anteroposterior view) (Figs. 4, 5, 6).

2. APA items and velopharyngeal port parameters

There was a significant negative correlation between the degree of velar mobility and each degree of open nasality and consonant imprecision, while a significant positive correlation between the size of the persistent gap and each degree of open nasality and nasal air emission Table 5.

3. APA items and gap distance during phonation

**Table 5:** Spearman’s correlation coefficient to study correlation between velar mobility, persistent gap and degree of open nasality, consonant imprecision and nasal air emission

	Velar mobility	Persistent gap
Degree of open nasality		
* r*	− 0.808*	0.436*
* p* value	0.001	0.033
Consonant imprecision		
* r*	− 0.589*	0.178
* p* value	0.002	0.405
Nasal air emission		
* r*	0.000	0.655*
* p* value	1.00	0.001

^*^Statistically significant

There was a significant positive correlation between the gap distance during phonation obtained from videofluoroscopy and each degree of open nasality, consonant imprecision, and unintelligibility of speech (Table 6).

4. Velopharyngeal port parameters and gap distance during phonation

**Table 6:** Spearman’s correlation coefficient to study correlation between gap distance during phonation and degree of open nasality, consonant imprecision, nasal air emission and unintelligibility

	Open nasality	Consonant imprecision	Nasal air emission	Unintelligibility
Gap distance during phonation				
* r*	0.669*	0.547*	0.064	0.494*
* p* value	0.001	0.006	0.767	0.014

*Statistically significant

There was a significant negative correlation between gap distance during phonation and degree of velar mobility, while a significant positive correlation between gap distance during phonation and the size of the persistent gap, Table 7.

**Table 7:** Spearman’s correlation coefficient to study correlation between gap distance during phonation and Velar mobility and Persistent gap

	Velar mobility	Persistent gap
Gap distance during phonation		
* r*	− 0.828**	0.501*
* p* value	0.001	0.013

*Statistically significant

## Discussion

Autologous cartilage was preferred to augment the posterior pharyngeal wall in this study to decrease destruction by host reaction. This agrees with Hess et al. [6], who used autologous or homologous costal cartilage to augment the posterior pharyngeal wall with a superiorly based pocket. Also, Desgain et al. [7] also recommended cartilage grafts and documented that cartilage implants can be well positioned and do not migrate downwards, unlike injected material. Used cartilage could be costochondral [7], tragal [8], septal and conchal [9].

In this study, conchal and/or septal cartilages were used according to the needed bulk measured by lateral view videofluoroscopy regardless of the patient’s age. This agrees with Maniglia and Maniglia [10], Bejar et al. [11], and Yilmaz et al. [12], who stated that using septal cartilage early in children is a safe procedure, provided that it is carried out carefully, with appropriate conservation of cartilage, and respect for facial growth centers. However, El-Rashidi et al. [9] used conchal cartilage in patients less than 18 years old and septal more than 18 years old.

Augmentation pharyngoplasty was done for patients having gaps up to 7 mm with good results so that it could be used in VPI post-adenoidectomy up to 7 mm. Although Dejonckere and van Wijngaarden [13], Denny et al. [14], Bluestone et al. [15], Sturm and Jacob [16], and Ulker et al. [17] stated that augmentation pharyngoplasty is suitable for cases with good palatal motion and velopharyngeal gaps that do not exceed five mm and that larger gap sizes facilitate the chance of implant failure. However, Furlow et al. [18] documented that in cases that Teflon has injected, speech outcomes of the 6 to 10 mm gap size group were better than the 0 to 5 mm group. In agreement, Lypka et al. [19] found that cases with significant gaps up to 15 mm have tolerated stacking of grafts well, with excellent speech outcomes.

On speech evaluation, perceptual improvement was observed postoperatively in obligatory speech disorders (degree of open nasality, nasal emission, and imprecision of consonants) and intelligibility of speech. Also, a significant positive correlation was observed between the size of the gap distance during phonation and the degree of open nasality, consonant imprecision, and unintelligibility of speech. However, there was no change in the compensatory speech disorders. Obligatory speech disorders could explain this due to structural disorders in the VPP. They are corrected by surgical intervention by posterior pharyngeal wall augmentation, while compensatory speech disorders are functional disorders and need speech therapy to be corrected. This is agreeable with Khafagy et al. [8], who found that cartilage pharyngoplasty improved the percent of speech intelligibility after surgery.

In post-operative videofluoroscopic assessment, there was a significant improvement in the degree of velar mobility, and 100% of cases showed coronal closure compared to 62.5% pre-operative. This revealed that cartilage pharyngoplasty appeared to have a facilitating effect on velar motility. Fifty percent of the patients showed grade 4 velar mobility within 1 month following the operation. This could be explained by the augmentation pharyngoplasty decreasing the gap distance and making the VPP and velar movement sufficient for complete closure. Also, this decrease in gap distance tends to encourage more excellent lifting action in the soft palate. Gray et al. [20] reported that the shape of the pre-operative gap, such as coronal or circular, did not seem to be a factor in predicting success or failure in his study.

Post-operative videofluoroscopic assessment for the gap distance between the velum and posterior pharyngeal wall significantly reduced the gap distance at rest and during phonation. Similar results were reported by Khafagy et al. [8] as they found that cartilage pharyngoplasty improved the grade of closure and decreased the velopharyngeal gap.

Posterior pharyngeal wall augmentation is a technique that is largely forgotten and understudied. Both autogenous tissue and exogenous implants were utilized for such approach [20]. Many trials were performed with various materials, such as paraffin [21], silicone [22], Teflon [18], collagen [23], calcium hydroxyapatite [24], fat [25], proplast [26], Gore Tex [19], and cartilage [27]. All these materials have advantages and disadvantages but none of them have been widely adopted [27]. Overcorrection is needed for all injectable augmenting material, because the vehicle solution is resorped [27]. There are many complications from implantation of foreign substances in the posterior pharyngeal wall including infections, extrusion, resorption and even migration of the material after insertion. Granuloma formation has also been associated with Teflon implantation. [28]. Also the implant may be inadequate in size or in the wrong place to totally occupy the gap. Conversely, overcorrection may happen that might cause hyponasality and obstruction of upper airway. Thus far, no alloplastic material has been found to be completely safe, effective, and reliable, but each autoplastic material has had its own long-term durability [29]. Few studies aimed at evaluating cartilage implantation in VPI. This method has.

a minimal complication rate because of autologous tissue application [7]. The present study revealed that posterior pharyngeal wall augmentation could be used in VPI post-adenoidectomy up to 7 mm, leading to better speech outcomes. Also, it revealed that using conchal and/or septal cartilage as a graft regardless of the patient’s age is a safe procedure. Using multiview videofluoroscopy to measure velopharyngeal gap distances gave valuable data for the teamwork to provide the bulk of the augmentation for correcting VPI.

## Conclusion

Posterior pharyngeal wall augmentation by autologous graft as cartilage is an effective surgical procedure regarding VPI post-adenoidectomy. Septal cartilages can be used as a graft regardless of the patient’s age, provided that it is carried out carefully, with appropriate conservation of cartilage and respect for facial growth centers. Also, with thickness up to 7 mm.

## Recommendations

Further studies are recommended with a more significant number of patients, different graft materials, and a more extended period of follow-up to determine long-term outcomes. The present study was more familiar with septal and auricular cartilage, harvesting they were selected as graft material. Practicewise, these donor sites had the disadvantage of two separate incisions and limited cartilage thickness, so rib graft is recommended as a better donor site.

## Data Availability

The data sets used and/or analysed during the current study are available from the corresponding author.
